# Permeable Concrete with Recycled Aggregates. Study of Its Mechanical and Microstructural Properties

**DOI:** 10.3390/ma18040770

**Published:** 2025-02-10

**Authors:** Miguel Á. González-Martínez, José M. Gómez-Soberón, Everth J. Leal-Castañeda

**Affiliations:** 1 Sección de Posgrado e Investigación, ESIA Z, Instituto Politécnico Nacional, Av. Luis Enrique Erro S/N, Unidad Profesional Adolfo López Mateos, Zacatenco, Alcaldía Gustavo A. Madero, Ciudad de México 07738, Mexico; mgonzalezm1509@alumno.ipn.mx; 2 Department of Architecture Technology, Barcelona School of Building Construction, Polytechnic University of Catalonia, Av. Doctor Marañón 44-50, 08028 Barcelona, Spain

**Keywords:** compressive strength, construction and demolition waste, flexural strength, permeability, reclaimed asphalt pavement aggregates, recycled concrete aggregates, SEM, TGA

## Abstract

The construction industry is a fundamental sector for the development of countries; however, it produces negative environmental impacts due to the demand for natural resources and the generation of construction and demolition waste (CDW). Therefore, the pursuit of solutions to recycle and reintegrate these wastes, which often accumulate in poorly regulated areas, becomes not only an environmental priority but also an opportunity to transform a problem into an advantage. Utilizing these residues contributes to reducing the pressure on natural resources, minimizes the environmental footprint of the construction sector, and promotes a more sustainable and responsible model that can serve as an example for future generations. The properties of recycled concrete aggregates (RCA) and recycled asphalt pavement (RAP) were determined in order to subsequently obtain the properties of different permeable recycled concrete (RPC) elaborated from a factorial design 2^3^ with these aggregates. The properties studied were workability, permeability, volumetric weight, compression uniaxial, and bending. Finally, they were studied and correlated with their matrix microstructure by means of TGA and SEM tests, which allowed determining the compounds contained in the various mixtures and their impact on physical–mechanical behavior. The results indicate that RCA and RAP are feasible alternatives for making porous pavements in pedestrian or light traffic areas when recycled aggregates of 3/4” size are included in their matrix, resulting in the optimum dosage of the M5 3/4” mix in this research, whose mechanical properties are: uniaxial compressive strength: 15.39 MPa; flexural strength: 3.12 MPa; permeability: 0.375 cm/s.

## 1. Introduction

Construction is an economic activity that favors the development of countries through the creation of infrastructure [1], as well as stimulating the productivity of other economic sectors [2]; therefore, current macroeconomic theory suggests that there is a significant correlation between the behavior of the construction sector and countries’ economic dynamics [1]. Although construction and the activities linked to it have a positive impact on the nation’s economy, they also lead to more pressure on the management of resources [3]. This has caused impacts on the environment due to the emission of greenhouse gases (GHGs) [4], degradation of the natural environment due to overexploitation of material stocks [5], and, above all, the disappearance of fauna and flora [6]. As a result of the environmental impacts and exacerbated by poor management of resources in construction, this sector is currently facing a continuing shortage of mineral resources [7].

Concrete is an essential material for the creation of conventional buildings; it is the combination of adequate quantities [3] of water, natural aggregates—from 55 to 80% of the total volume [8]—and the addition of some of the different types of Portland cement. Cement is essential for producing concrete, and its production is considered to generate around 5% of all anthropogenic carbon dioxide (CO_2_) emissions worldwide [9] while consuming 7% of all energy in the industrial sector (with a foreseeable increase of 12 to 23% by 2050) [10].

To make matters worse, in a rehabilitation, construction, and demolition process, the so-called construction and demolition waste (CDW) is generated, which is made up of concrete, bricks, tiles, bituminous mixtures, gypsum, wood, glass, metals, plastics, solvents, earth, and sometimes even asbestos [11]. CDW that is not processed in recycling plants is dumped—in the best of cases—in landfills, which are not a suitable process for partial or final disposal [12], sometimes even reaching between 25 and 45% of their maximum admissible capacities [13]. This type of waste represents 50% of the total solid waste generated annually worldwide [14]; it has been concluded that after several recovery processes [15], it can be recovered by reincorporation, for example, in construction and paving works, by substitution or in combination with the usual aggregates [16]. The closed loop that includes the recycling of CDWs does not always result in a complete benefit for the environment since, within this cycle, the transport of waste and recycled aggregates (RA) will generate extra environmental impacts [15]. Therefore, it is necessary to reduce this waste in the first instance, thus also reducing the demand for virgin mineral resources, the consumption of fossil or renewable materials, the generation and consequent dragging effects of leachate, and the production of GHGs [16]; and leaving as a subsequent or second alternative of action, the promotion of those wastes that are not feasible to avoid

The treatment of CDWs for possible application as RA includes several stages, the first being the transport of the CDW to the transfer site or recycling plant. Here, it is classified through mechanical or manual procedures that allow the sorting of unsuitable solid components such as wood, paper, plastic, and metals, among others. As a result of the previous process, they are subjected to crushing [16] by diesel or electricity-operated crushers with different efficiencies and with variations in the resulting product if their effect is of the jaw or mill type. This treatment leads to the creation of new second-generation materials, such as recycled concrete aggregates (RCA) or recycled asphalt pavement (RAP), among others. In general, these new materials are called RA and, after being sieved according to the commercial sector standards, can be used in new applications.

As a result of the previous recycling process and the constitution of their matrix, the RCA have an excess of mortar adhering to the original aggregate and significant microcracks throughout their surface [17]. This adhered mortar is characterized by being rough and porous, causing the RCA to have a greater interfacial transition zone (ITZ) and absorption if compared to a natural aggregate (NA). This will eventually cause different or reduced benefits [18]. Currently, two treatment methods have been prescribed to improve RCA with these characteristics, seeking to reduce the amount of adhered mortar through different procedures such as mechanical treatments or the use of acid [19].

AP, on the other hand, is the term given to the gravels from removed pavements containing stone aggregate and asphalt for incorporation into new projects, which are generally asphalt pavements [20]. RAP can be recycled using three different methods: hot recycling (165 °C), warm recycling (100 °C), and cold recycling (mixing of removed pavement with a new binder) [21]. The use of RAP in new projects helps to reduce the demand for AN, reduces energy consumption, and thus considerably reduces CO_2_ emissions [22].

Given the sustainability of the substitution of NA by RA and the possible benefits of its use in the manufacture of pavements with low capacities or mechanical performance, such as street sidewalks, parking lots, and alleys [23], this second-generation material seems ideal for use. The variations in the mechanical properties of recycled pavements with RA have been the driving force behind research that seeks to improve their properties through various techniques and elaboration methods, with several alternative components such as silica fume [24], fly ash [25], blast furnace slag [26], and pumice powder with nanoclay [27] being researched.

There are several types of recycled concrete that can be manufactured using RA; however, the most sustainable is recycled permeable concrete (RPC), which is characterized by having an interconnected porous network that allows water to flow through its porous mesh structure [28]. Among the main benefits of this type of concrete, it is worth highlighting that when it is used as pavement [29], it favors the recharge of underground aquifers through rainwater runoff. It can also reduce the effects of heat islands in cities, as demonstrated by the adjustment of temperature and surface humidity, and it has a high capacity for noise absorption [30,31]. However, this cannot be guaranteed on a continuous basis due to a lack of maintenance [32].

Therefore, in this research, the behavior of recycled porous pavements was studied by replacing 100% of NA (by volume) with 50% of RAP (of sizes 3/8” and 3/4”) and 50% of RCA (3/8” and 3/4”), in addition to the use of silica sand, when required, plus cement and water. The different mixtures studied were established by means of a factor analysis 2^3^, developed in the Minitab 19.1.0.1 program (Philadelphia, PA, USA), selecting as variables the water/cement ratio (W/C), the percentage of fine aggregates, and the percentage of pores. Their respective values were W/C = 0.26 and 0.35, fine aggregates = 0 and 10%, and porosity = 15 to 20%, in accordance with the American Concrete Institute standard 522R-10 [33].

Prior to the dosage of the necessary amounts of RA, they were characterized in their physical properties according to the regulations; then, the dosage for making the RPC mix was established. The resulting specimens were tested in duplicate to establish the properties of compressive strength, flexural strength, and permeability. Finally, the RPC mixtures were analyzed by thermogravimetry (TGA) in order to relate their properties with the structure of their matrix, using Scanning Electron Microscopy (SEM) to identify their microstructure.

## 2. Materials and Methods

### 2.1. Materials

**Figure 1:**
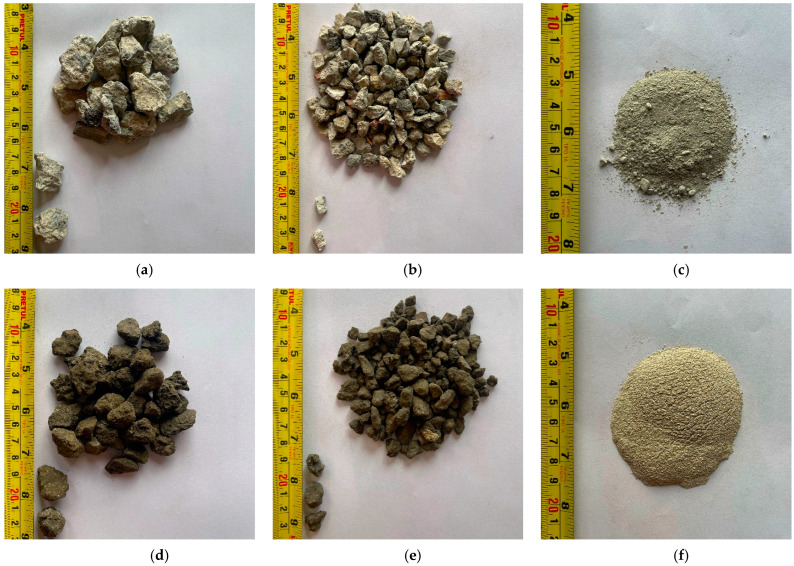
Matrix components of the RPCs under study. (**a**) 3/4” RCA; (**b**) 3/8” RCA; (**c**) Cement CPC 30R; (**d**) 3/4” RAP; (**e**) 3/8” RAP; (**f**) Silica sand smaller than sieve no0.

For the manufacture of the RPC specimens, Portland cement type CPC 30R was used, which complies with the NMX-C414-ONNCCE-2017 [34] standard, potable water according to the specifications of the NOM-127-SSA1-2021 [35] standard, silica sand smaller than that retained by the #30 sieve (ISO, 0.6 mm opening) and two different types of recycled coarse aggregates: RCA of 3/8” and 3/4” sizes and RAP with 3/8” and 3/4” sizes; both were donated by an RA treatment company. Figure 1 shows the solid components that will be integrated into the manufacture of the RPC, showing their typology, shape and grain distribution.

### 2.2. Methods

#### 2.2.1. Physical–Mechanical Characterization of Coarse RA

The characterization tests applied to the coarse recycled aggregates (3/8”, 3/4” RCA, and 3/8”, 3/4” RAP) were performed after selective sampling based on three basic samples for each size of study RA, which were then used in each of the assays, as well as to establish the average value for each of the determined properties and its standard deviation. Table 1 shows the property of the study and the applicable regulations; when acceptance limits are prescribed, these are also indicated.

The results of the tests carried out to determine the physical–mechanical properties of the ARs used in this research are shown in Table 2 and Figure 1. In this table, it can be seen that the aggregate with a higher density is the 3/4” RAP due to the layer of asphalt covering it; its treatment process does not remove this material since the purpose of using the RAP is to apply it in low-density road projects. However, not all this material is used, with some accumulated remnants.

The absorption percentage of the 3/8” and 3/4” RCA was very similar to the values recorded by Özçelikci and Şahmaran (2023) [41]. These absorption values are associated with its structure, which is composed of aggregates and mortar, elements that lead to a series of different types of pores (micropores (1 to 100 nm), mesopores (100 nm to 0.01 mm) and macropores (0.01 mm to 1 cm)) [42,43], and the resulting microcracks produced during the crushing process. On the other hand, the 3/8” and 3/4” RAPs had an absorption percentage below 2%, in this case, despite the materials having pores or micro-fissures, as the entire surface of the aggregate is covered by a layer of asphalt that limits the access of water to its interior [43].

The volumetric weight of RAs is also affected by the size of the particles and the materials they are composed of; the smaller the particles are, the more pores or defects —micro-fissures—they will have. Both characteristics are a consequence of their crushing process, thus causing a decrease in the weight of the RA [43].

Based on the stipulations of the Mexican Institute of Transportation (IMT) [44], the typical value of the CDVW of the RAP is in the order of 1600 to 2000 kg/m^3^, which establishes that the 3/4” RAP is ideal for use in mixtures, while the 3/8” RAP has a high number of pores or microcracks. Therefore, its CDVW is lower than the typical values but without reaching the extreme RCA values of 3/8”.

**Figure 2:**
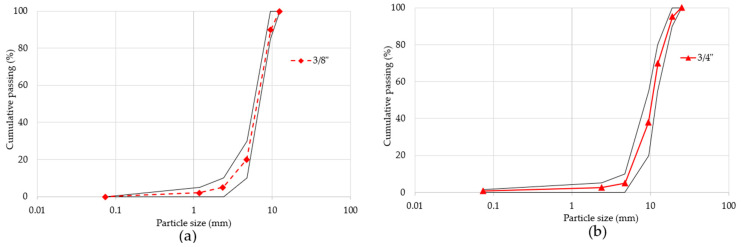
Particle-size profile of RA used in the development of the RPC mixes. (**a**) 3/8” RA; (**b**) 3/4” RA. The continuous black line shows the maximum and minimum acceptable limits according to regulations [40].

The RCA and RAP RAs used in this research do not present an adjusted or ideal granulometry profile, as they exceed the limits of the NMX-C-111-ONNCCE-2018 standard [40]; therefore, it was decided to create an adjusted particle-size profile. To achieve the above, with the 3/8” and 3/4” RCA and RAP aggregates, an adjusted particle-size curve was designed (Figure 2), the composition of which allowed the different mixture designs to be correctly prepared.

#### 2.2.2. Statistical Design of the Experiment

The specification of the types of mixtures for an adequate statistical analysis was carried out by means of a complete two-level factorial design with three factors (2^3^). Its determination was obtained using the Minitab 19.1.0.1 program (Philadelphia, PA, USA). The factors were determined after considering the most significant of the specifications included in the American Concrete Institute (ACI) 522R-10 permeable concrete guide [33], which are presented in Table 3.

#### 2.2.3. Mix Design

The result of the design of the mixtures was carried out using a factorial design 2^3^, which resulted in a total of eight variants (Table 4), which were made in duplicate for each size of RA (3/8” and 3/4”).

The nomenclature used in each variable was MX-XX”, where the first letter “M” refers to the mixture, the first X refers to the mixture number (which ranges from 1 to 8), and the nomenclature X/X” refers to the size of the aggregate (3/8” or 3/4”) used in the mixture. RPC mixtures from M1-X/X” to M4-X/X” refer to mixtures made without silica sand, while mixtures from M5-X/X” to M8-X/X” refer to mixtures containing 10% silica sand.

#### 2.2.4. Composition of RPC Mixtures

Based on the different combinations obtained and the properties of the recycled coarse aggregates, all the necessary RPC mixtures were dosed in accordance with the ACI 522R-10 guideline [33]. Here, 100% of the required volume of coarse aggregates was replaced with 50% of RCA and 50% of RAP.

Table 5 shows the quantities required in kilograms for 1 m^3^ of RPC for the different mixtures with 3/8” and 3/4” RA.

#### 2.2.5. Preparation of RPC Specimens

The preparation of the cylindrical specimens and the RPC beams to be used in determining the properties of simple compression and three-point bending were carried out following the methodology prescribed by the ASTM C192/C192M-15 standard [45]; for this, cylinders of Φ = 100 mm in diameter and 200 mm in height were modeled for testing at simple compression, as well as beams with dimensions of 150 × 150 × 500 mm for three-point bending tests. After the specimens were molded, 24 h later, they were extracted from the molds and then immersed in water for curing, where they remained until they reached the age of their established test (7 and 28 days).

#### 2.2.6. Characterization of RPC

The RPC specimens subjected to compression tests were tested in a TREVIOLO model YIMC109NC compression press (MATEST, Bergamo, Italy), keeping to the specifications of the ASTM C39/C39M-23 [46] standard, and setting the applied load gradient at 0.40 MPa/s. For this test, the cylinders were previously prepared, which involved facing the upper part of the cylinder (concreting direction) by covering it with a thin layer of mortar in order to guarantee better load distribution; likewise, the cylinders were covered on both sides with molded neoprene pads (ASTM C39/C39M-23 [46]).

For the bending test, the guidelines of the ASTM C42/C42M-04 standard [47] were applied; a load supported at three points of the specimen was applied to each test specimen to produce the three-point bending effect; the test was performed using a Tinius Olsen 23,471 press equipment (Testing Machine Company, Philadelphia, PA, USA).

**Figure 3:**
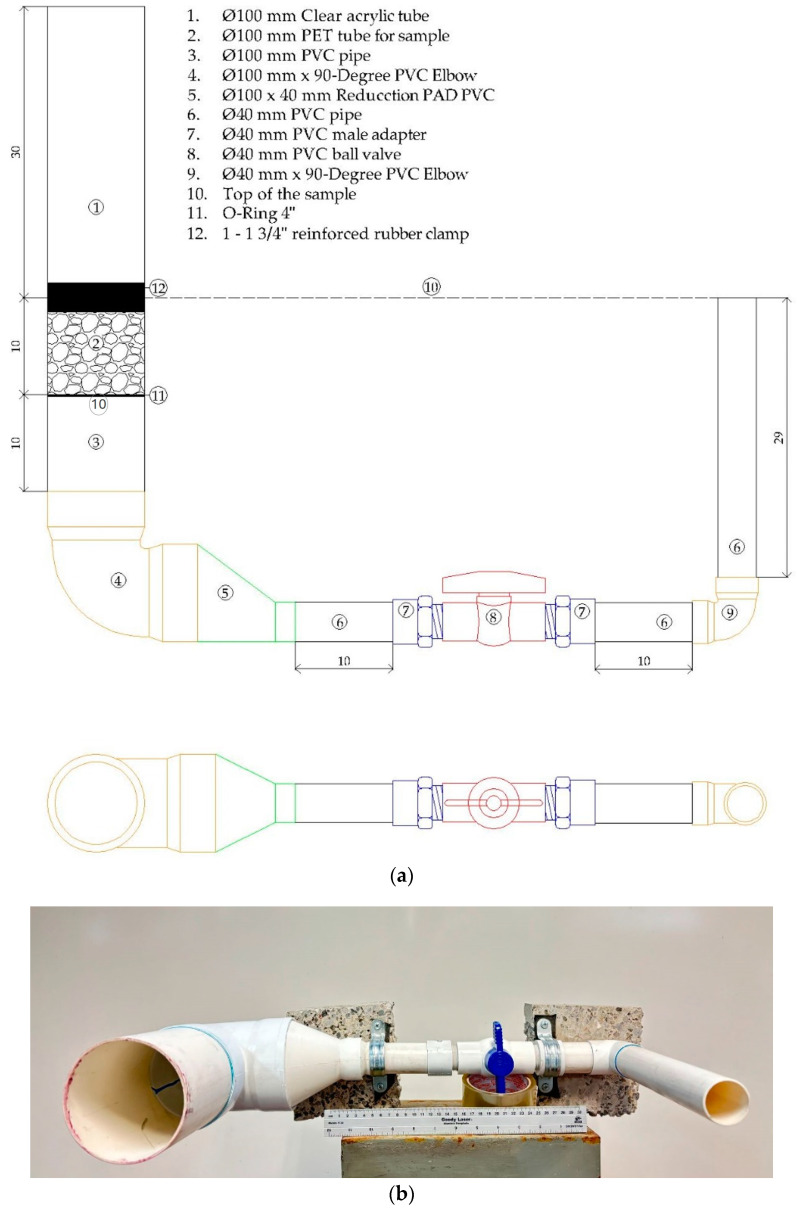
Permeameter prototype developed for permeability testing. (**a**) Prototype schematic (dimensions in cm); (**b**) Permeameter.

For the permeability test (determination of the permeability constant k) (Figure 3) cylindrical specimens with a Φ = 100 mm and a height of 100 mm were used following the ACI 522R-10 standard [33]; for this, a permeameter was manufactured with PVC pipes and an acrylic tube of Φ = 100 mm (Φ internal) and 300 mm in height (with a capacity of 2356.20 cm^3^) (Figure 3). Each test specimen was positioned inside the PVC pipe, and the acrylic tube was arranged above them; a rubber gasket was used to prevent leaks between the PVC pipe and acrylic pipe connection. The test procedure involved carrying out the following phases: verifying that the water shut-off valve was in the closed position, filling the acrylic tube to its full capacity with water, opening the shut-off valve, and timing how long it took the water to travel through the entire circuit. The travel time was established using a standard H-5670 chronometer (ULINE, Apodaca, Nueno León, Mexico).

#### 2.2.7. Methodology for Thermogravimetry Analysis (TGA)

**Figure 4:**
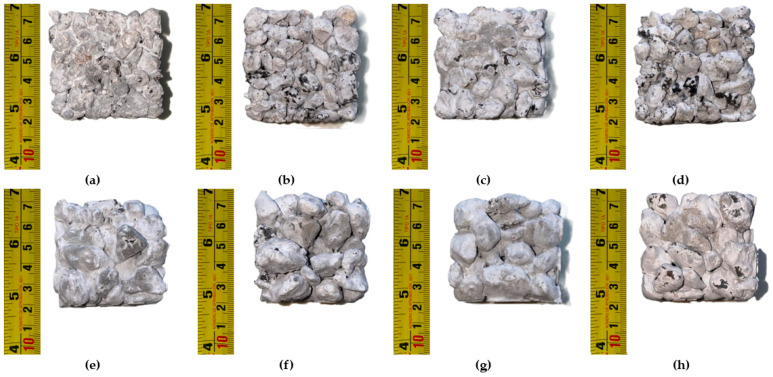
50 × 50 mm. cubes of the selected RPC mixtures for the TGA study. (**a**) M1 3/8”; (**b**) M4 3/8”; (**c**) M5 3/8”; (**d**) M7 3/8”; (**e**) M1 3/4”; (**f**) M4 3/4”; (**g**) M5 3/4”; (**h**) M8 3/4”.

For the TGA analysis, samples M1 3/8”, M4 3/8”, M5 3/8”, M7 3/8”, M1 3/4”, M4 3/4”, M5 3/4” and M8 3/4” (Figure 4) were selected for their importance in the results obtained in the simple compressive strength tests. The design of the selected mixtures was replicated in the manufacture of 50 × 50 mm cubes, which were later used in the TGA test.

**Figure 5:**
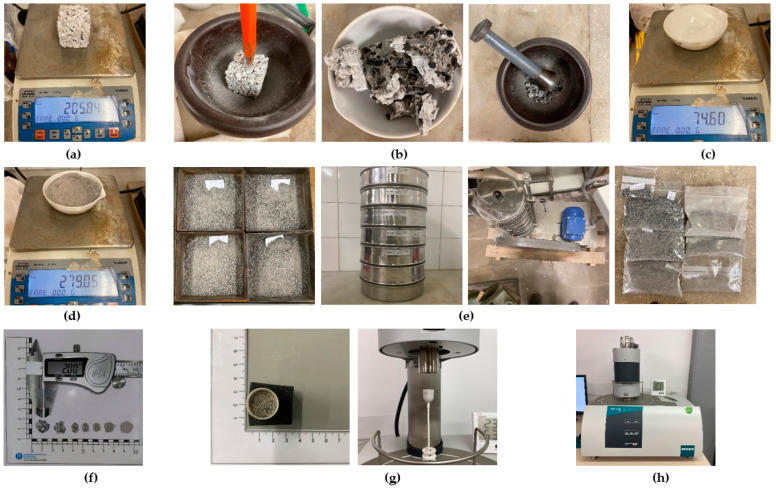
Procedure for TGA analysis. (**a**) Determination of the weight of RPC specimens; (**b**) Manual crushing of RPC specimens; (**c**) Determining the tare weight of the capsule; (**d**) Determination of the weight of the crushed material; (**e**) Screening and sieve size separation; (**f**) Representative reconstituted sample of 3.5 g; (**g**) Preparation for TGA analysis; (**h**) In process TGA analysis.

After curing, the RPC cubes were carefully crushed manually using a metal mortar capsule, a chisel, and a steel mallet. Each crushed sample was then sectioned by particle size according to the indications of the UNE-EN 12192-1 [48] standard in the following fractions (mm): 2, 1, 0.5, 0.250, 0.125, 0.063 and the remainder that passes the #200 mesh sieve (0.074 mm). Finally, a recomposition was performed on a proportional scale to obtain a representative sample of 3.5 g. The detailed procedure is shown in Figure 5. For the TGA test, STA 449 F5 *Jupiter* (NETZSCH, Selb-Alemania, Germany) equipment was used, programmed to apply two temperature ramps during the course of the test, the first of them between 21 and 500 °C with an increase of 3 °C/min.; and the second between temperatures of 501 and 1000 °C, with an increase of 2 °C/min. The latter was performed more slowly to be able to better capture possible changes or variations in the mass in the samples tested.

Once the TGA results were obtained, the derived percentage curve (% dTG) was generated, and a simple moving average with a base of 7 (Equation (1)) was proposed, as this value was the one that best fit the behavior of the differential thermogram curves.

(1) SMA7=∑in=Xi+…+Xnn

where: ${SMA}_{7}$ = simple moving average and n = 7, each individual value (X_i_) represents the percentage difference in weight loss between the consecutive measures of TGA.

To complement the analysis, the partial and total areas under the curve were compared [49]; that is, the percentage of weight loss versus the increase in temperature. For this process, two values of the percentage of weight loss (W) and their respective temperature increases (T) were used to calculate the area under the desired curve. Equation (2) was used.

(2) $$\int_{0}^{i}f\left(x\right)dx=\left(t_{2}-t_{1}\right)\left[\frac{W_{1}+W_{2}}{2}\right]$$

where: f(x) (%-°C) is the area under the curve; t_1_ (°C) is the initial temperature of the pair of values studied; t_2_ (°C) is the final temperature of the pair of values studied; W_1_ (%) is the initial percentage of weight loss of the pair of values studied; and W_2_ (%) is the final percentage of weight loss of the pair of values studied.

## 3. Results and Discussion

The results of the slump, simple compression, flexion, volumetric weight, and permeability tests are shown in the following sections and figures, demonstrating the adequate degree of general compliance with the values presented, as they are positioned within the parameters established by ACI 522R-10 [33].

### 3.1. Slump of RPC

**Figure 6:**
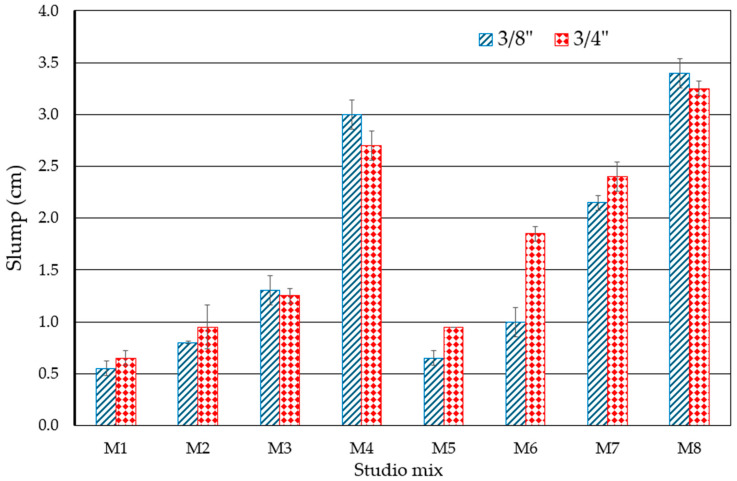
Results of slump tests on RPC mixes. Note: Slump limits = 2.00–0.00 cm [50].

The slump of the RPC mixtures is shown in Figure 6, showing how the W/C ratio can affect their behavior, causing, for example, in cases of W/C = 0.35, the mixtures to flow more and even exceed the acceptable slump limits [50], as evident in the M4 and M8 samples. However, in general terms, variables with a W/C = 0.26 do not do so, for example, in the M1 and M5 samples. Regarding the size of the particles studied (3/4” and 3/8”), it has been shown that the stone structure of the concretes (their particle sizes, their distribution, etc.) affects the behavior of the slump [51]; in this work, the samples with a larger particle size (3/4”) improve the increase of their slump in general terms. Finally, the results of the samples are divided into two large groups related to each other: those whose matrix does not include the fine aggregate of silica sand (particles < sieve #30), which includes samples M1 to M4, and those whose matrix includes 10% of the fine aggregate of silica sand (particles < sieve #30), which includes samples M6 to M8. In both cases, the increase in the design porosity percentage of the mixtures is directly correlated with the increase in the slump; this can be understood if it is considered that these holes facilitate the mobility of the mixture particles in their fresh state [52].

### 3.2. Compressive and Flexural Strength

**Figure 7:**
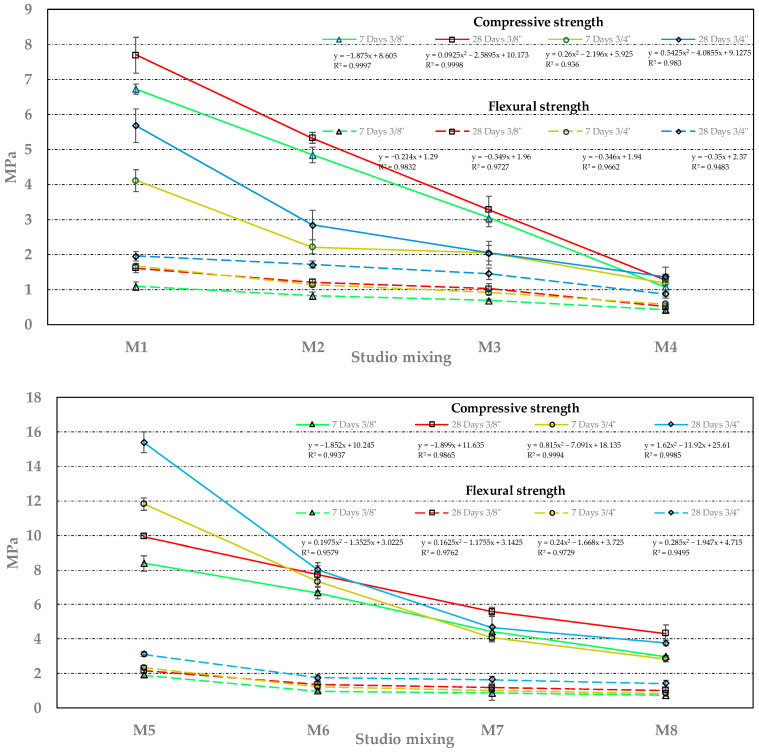
Compressive and flexural strength of the RPC mixtures studied. Note: Compressive strength limits = 3.5–28 MPa [50], Flexural strength limits = 1–4.8 MPa [50].

The compressive and flexural strength achieved by the different RPC mixtures is presented in Figure 7. Here, a similar behavior pattern is observed if they are grouped in the mixtures in M1 to M4 with respect to the grouping of M5 to M8. This pattern describes how the variables of the factorial design affect resistance. For the first of the groupings (M1 to M4), a lower development in resistance can be seen compared to the second grouping (M5 to M8), which is −49% in compression and −25% in bending (always considering the average values of all the samples studied). As the latter grouping contains silica sand, it gives them greater resistance. On the other hand, for both groups, the increase in porosity leads to a reduction in resistance; specifying a porosity of 15% to 20% leads to a reduction of −58% in compression and −42% in bending. Finally, when porosity is a constant (along with the other variables), and the W/C ratio is modified, the W/C change from 0.26 to 0.35 causes a modification of the results since a higher W/C ratio leads to a reduction in strength (cement hydration capacity) which results in a loss of −36% in compression and −33% in flexion.

In terms of strength capacity, the results of the compression tests of the RPC with RCA and RAP RA show low strengths, both for compression and bending (see Figure 6). The M5 3/4” is the only mix that exceeds a higher compressive strength of 10 MPa (15.39 MPa). However, it is true that in this group of samples containing silica sand, the minimum requirement established by the IMCYC [50] (3.5 MPa) is satisfied. In terms of flexural strength, for the M5 to M8 grouping, the minimum acceptable limit—1 MPa—is also practically satisfied (only M8 is very close to satisfying it); while only M1 satisfies it in the other group.

As for the numerical regressions selected to describe the behavior of the different samples (an R^2^ ≥ 0.9 was established as a requirement), and for the M1 to M4 grouping, the equations are mostly either inverse linear, which establishes the proportionality of the change in the variables studied, or inverse second-degree polynomials with a reduced coefficient of the term x^2^, which could show the existence of a reduced variation effect in the behavior of its compressive strength that was not established in factorial design 2^3^ of this research. On the other hand, the M5 to M8 grouping generally establishes second-degree polynomial equations, indicating that this grouping has interaction between its complex or variable components in its material matrix structure, thus allowing optimal behavioral conditions to be established in some of its variables.

With regard to the degree of maturity that the mixtures reach, it can be seen that the behavior is typical of conventional concrete. It is noteworthy that, of the mixtures studied in this research, the variables that reach higher values of compressive strength are also those that have greater strength gain from 7 to 28 days.

Comparing the previous results obtained, it is established that these reached values lower than those reported, for example, by Pérez-Ramos (2009) [53] (despite both studies having similar mixture designs). However, in both cases, the compressive and flexural strength of the RPC is affected by the increase in the percentage of pores or cavities (from 15 to 20% in the case of this research). Another factor that affected the strength of the different mixtures was the type of aggregate; as explained by Brasileiro et al. (2024) [54], the RAs have microcracks, pores, and excess mortar, which negatively affects the strength of the concrete. They also mention that the quantity and quality of the cement paste influence this mechanical characteristic due to possible adhesion to the aggregate. The authors mention that RAs can generate greater resistance if they are small; however, this research shows that aggregates of 3/4” developed greater compressive and flexural strength. Thus, the strength depends on the contact area between the RAs, as well as the quantity and quality of the cement paste.

### 3.3. Permeability and Volumetric Weight

**Figure 8:**
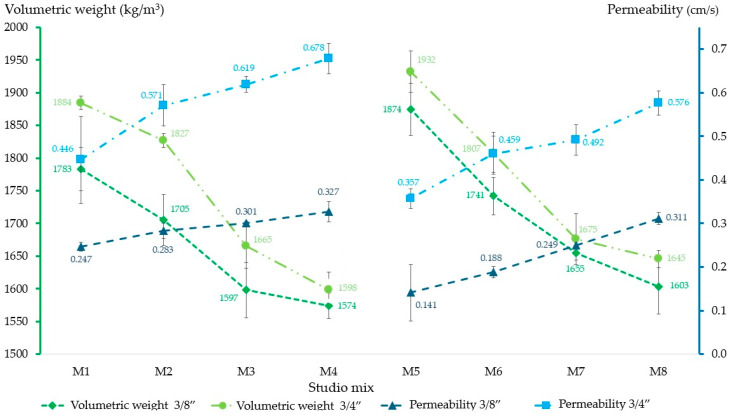
Volumetric weight and permeability of RPC mixtures with 3/8” and 3/4” RA. Note: Volumetric weight limits = 1600–2000 kg/cm^2^ [50]; Permeability limits = 0.20–0.54 cm/s [50].

When analyzing the results of permeability and volumetric weight in Figure 8, it can be seen that these physical properties are inversely proportional: when permeability is high, volumetric weight is lower. On the other hand, the permeability index in the M5 3/8” and M6 3/8” mixtures is below the limit established by ACI 522R-10 since the pores that allow the passage of water are closed or obstructed. In general terms, the study samples establish permeabilities within the permitted range—0.20 to 0.54 cm/s—, but when comparing mixtures that include 3/8” RA with those that use 3/4”, the latter present a degree of filtration that is twice as much as the former. The permeability index is also affected by the quality of the cement paste since, in mixtures where silica sand was used—M5 to M8—the cement paste produced by the sand significantly reduces or closes the pores, causing lower filtration rates.

The volumetric weights of the different RPC mixtures are equivalent to the theoretical value resulting from the sum of the elements that constitute them; however, the M3 and M4 mixtures with 3/8” RA have a volumetric weight lower than that accepted by regulations [50], although these values are close to the limit.

### 3.4. Analysis of Factorial Design 2^3^

Table 6 shows the values of the variables or factors of study (T) and their significance (*p*) obtained in the factor analysis processed in the Minitab 19.1.0.1 program (Philadelphia, PA, USA). It can be seen in this table how the factors used in the design are not significant on their own (*p* ≤ 0.05), but in combination with other factors or with the three factors as a whole, they show their significance in certain properties of study (indicated in bold in the table). In general terms, the mixtures of 3/8” RA present more statistically significant correlations than the RA 3/4”. The best for both groups of RA sizes are as follows: for 3/8”, the combination of factors 2 T (W/C *% Empty Ratio) with a *p* = 0.927, while for 3/4”, the combination of 2 T factors (W/C *% Fine Ratio) with a *p* = 0.984. Likewise, at the end of each column of properties, the value of the coefficient of determination of each test is observed, with the square root of this factor being equal to Pearson’s correlation coefficient (value equivalent to R^2^), all of which could be considered to be positive correlations with a magnitude or strength of correlation classified as strong or very strong (depending on the classification considered [55]).

**Figure 9:**
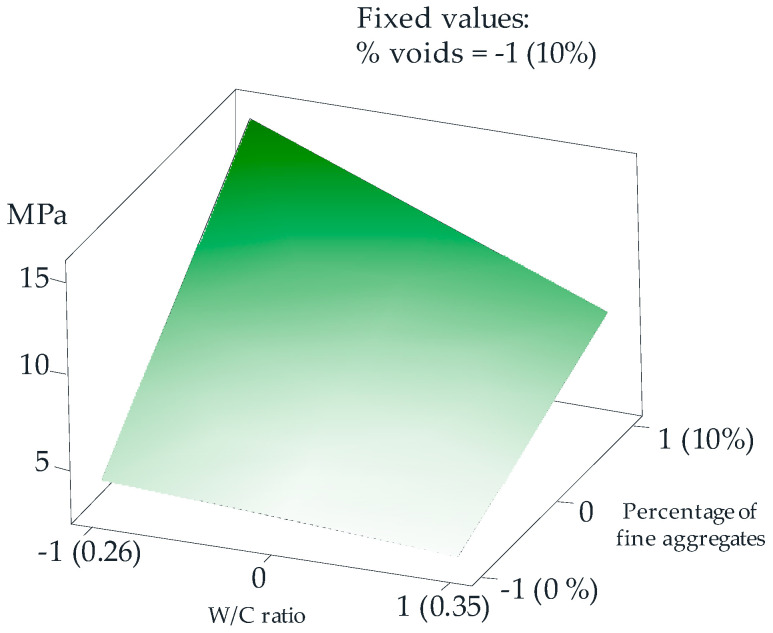
Compressive strength response surface with 3/4” RA.

As the main objective of this research is to find an optimal mix design for maximum compressive strength, the most statistically significant results were established for 3/4” RA when the percentage of voids was kept at the low value, 15% (value assignment −1). With this value assigned and fixed, a surface graph was obtained, which shows that the factor of the W/C ratio = 0.26 (value assignment −1) and the 10% of fines (assignment of value 1) are optimal for achieving the highest feasible resistance. Figure 9 graphically demonstrates the above and shows the behavior of the compressive strength when the values of the factors of the W/C ratio and percentage of fine aggregate are not at their optimal point.

With this analysis, it can be stated that the properties of the mixtures designed in this research can be established with their respective equation by means of a linear regression analysis. However, the results of the permeability tests may vary since this is a factor that is also linked to other variables such as the shape of the aggregate used, the type of compaction applied—manual or mechanical—and the vibration applied at the time of placement.

### 3.5. Thermogravimetry Analysis (TGA)

**Figure 10:**
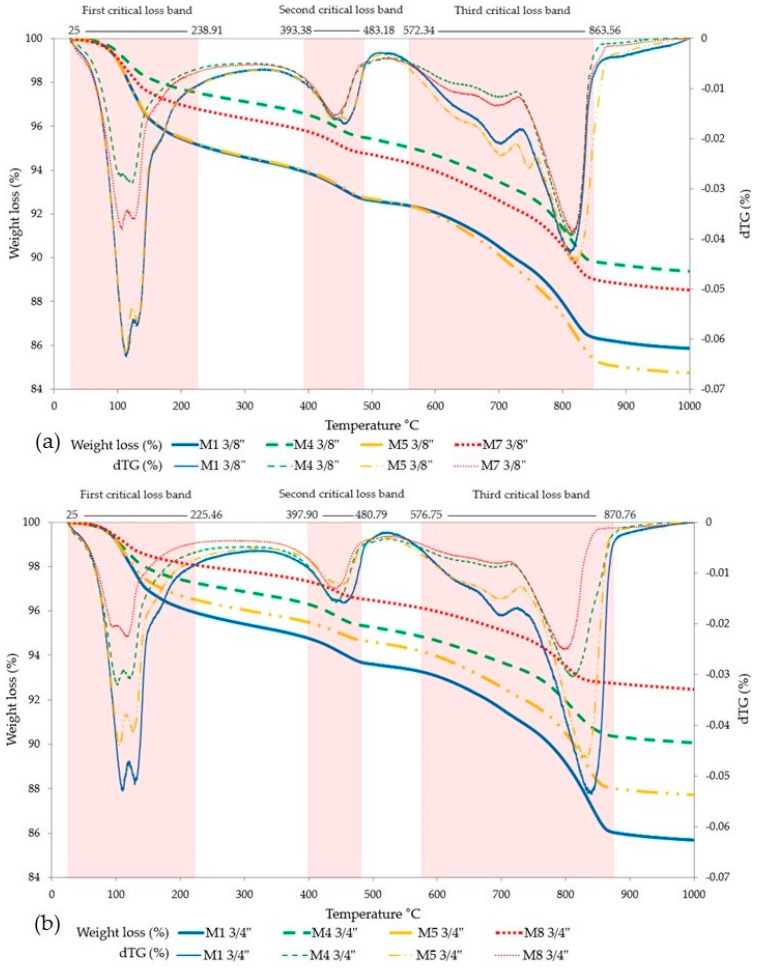
Weight loss vs. temperature increase. (**a**) 3/8” RA mixes; (**b**) 3/4” RA mixes.

The TGA results (Figure 10) indicate a weight loss caused by the calcination of the elements composing the matrix total of the RPC (RCA + RAP + Cement + Silica sand—when it was included). This weight loss ranges between 10% and 15% for mixtures with 3/8” RA, while for mixtures with 3/4” RA, it varies from 7% to 14%. In the tests, the weight loss behavior due to calcination of the material shows similar curves, in which three critical loss bands are identified, with the third band being the longest (with a bandwidth that begins at a temperature of 570 °C and ends at 870 °C). It should be noted that although there are three critical weight loss bands, weight losses are evident throughout the test process, from 25 °C to 1000 °C. To establish exactly where each critical loss band begins and ends, the angle of the slope was determined for every three pairs of continuous thermogram data, determining that the temperature range that produces slopes between −0.5° to −0.4° shows how far each one extends.

**Figure 11:**
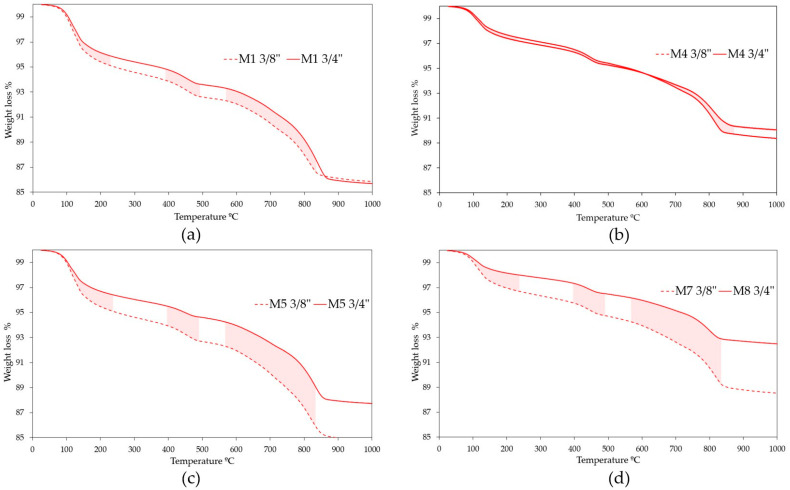
Comparative thermogravimetric analysis. (**a**) M1 3/8” vs. M1 3/4”; (**b**) M4 3/8” vs. M4 3/4”; (**c**) M5 3/8” vs. M5 3/4”; (**d**) M7 3/8” vs. M8 3/4”.

After obtaining the results and comparing the weight loss and area under the curve of the mixtures 3/8” RA against the mixtures 3/4” RA (Figure 11), it was determined that the weight loss is greater when 3/8” RA is used. This shows that the choice of mixtures with 3/4” RA as optimal, through analysis of the factorial design 2^3^, is consistent with the structure of their matrices being more stable at increases in temperature. Therefore, they are more resistant (but only if the size of the RA is taken into account as an incidence factor). However, these differences are greater when silica sand is added to mixtures of RPC with RA, which can be directly attributed to the main component of the sand (silica). In numerical terms, the comparison of the mixtures (M1 3/8” vs. M1 3/4”, M4 3/8” vs. M4 3/4”, M5 3/8” vs. M5 3/4” and M7 3/8” vs. M8 3/4”) establishes a difference in weight loss of more than 10% for each comparison of the cases studied.

**Figure 12:**
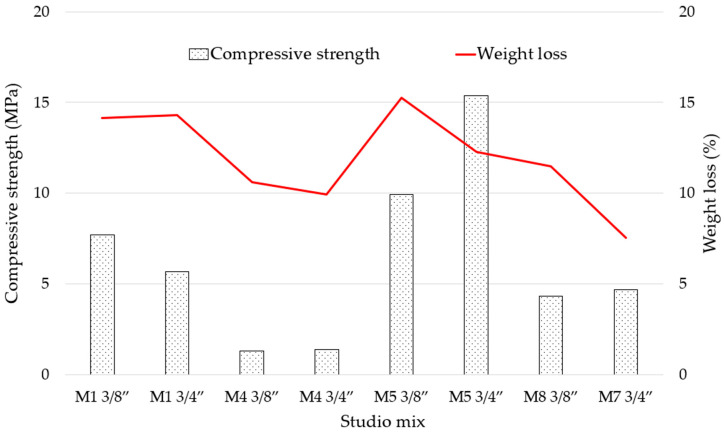
Compressive strength (MPa) vs. Weight loss (%) in TGA for RPC mixes.

As shown in Figure 12, the lower percentage of weight loss is linked to low compressive strength; however, a higher percentage of weight loss due to calcination of the components does not establish with certainty a better compressive strength since higher compressive strength results are also the variables that established the values of material loss by calcination at greater than 10%.

To determine the compounds or materials in the matrix that induce the mechanical behavior of the different mixtures studied, the details of elements that are oxidized, eliminated, or susceptible to being affected by the increase in temperature—from 25 °C to 1000 °C—were grouped (Figure 11).

**Figure 13:**
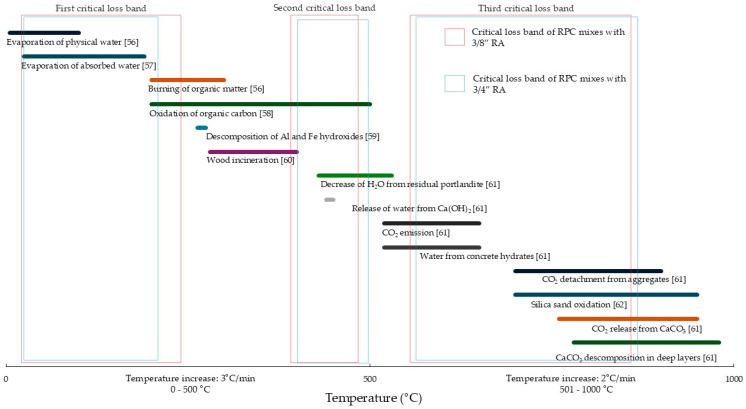
Temperature ranges in which the different elements volatilize. Figure made from previous research [56,57,58,59,60,61,62].

The mechanical behavior of the RPC mixtures depends to a large extent on the different compounds that compose the matrix; evidently, the most-found compound is water (H_2_O) since, according to the diagram in Figure 13, it is included in different layers. Likewise, in this diagram, it is observed that not all the elements that are volatilized are located in the critical loss bands; some of them, such as wood and organic matter, are found outside. Nevertheless, in most cases in which an RA is used, traces of wood and plastics, among others, are likely to be found.

Within the group of critical loss bands, the most significant is the third since it extends over a range of more than 300 °C. In this band, it can be seen that weight loss is linked to a greater degree of significance with CO_2_ gas emissions due to the decomposition process.

**Figure 14:**
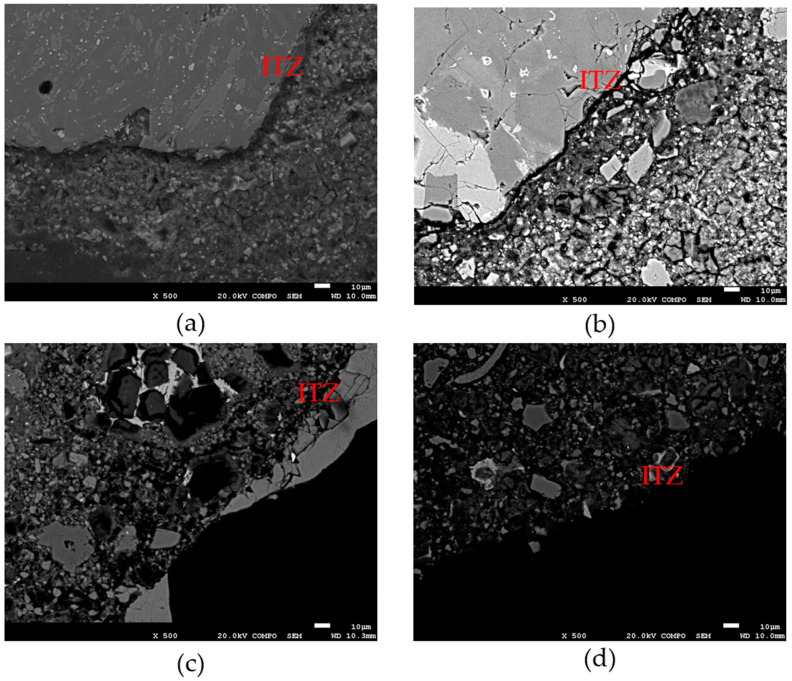
ITZ of the RPC samples studied by SEM. (**a**) M4 3/8”; (**b**) M5 3/8”; (**c**) M4 3/4”; (**d**) M5 3/4”.

Finally, to validate the above, it was necessary to observe, using the SEM technique, the matrix of the samples that developed greater compressive strength—M5 3/8” and M5 3/4”—versus those with lower resistance—M4 3/8” and M4 3/4”—. Figure 14 shows the interfacial transition zone (ITZ) of the samples of these mixtures, in which it can be seen how the cement paste integrates with the RA. The surface of the aggregate in question allows greater or lesser adherence with the cement paste, according to the relief of the surface and the thickness of the ITZ. In the case of M5 3/8” and M5 3/4”, the ITZ is of reduced thickness, with a continuous transition and free of excessive porosity; while in the case of the M4 3/8” and M4 3/4” samples, it is thicker, with non-continuous transition and greater porosity. This directly relates ITZ to the ability of RPCs to achieve better mechanical properties (e.g., compressive strength), establishing the axiom that when ITZ is thinner, continuous, and low porosity, better mechanical capacities will be induced in RPC.

## 4. Conclusions

In this study, it is established that recycled aggregate (RA) can be a real and feasible option for use as an aggregate in the composition of permeable recycled concrete (RPC) mixtures. However, it has also been demonstrated that specific considerations must be considered when selecting these materials due to their inherent characteristics, such as microcracks, excessive porosity, and remnants of other materials, which reduce the effectiveness of RA. These issues must be adequately addressed through treatments such as mechanical processes or acid washing, which could further improve the performance of RPC.

The optimal RPC mixture achieved its characteristics not only due to the ideal combination of studied variables but also because of the size and shape of the aggregate, which contributed to greater rigidity. When the contact surface is larger and rougher, the mechanical properties of these concretes are enhanced.

The M5 3/4” mixture emerged as the most outstanding in this study. Its properties were the best among the mixtures analyzed, a conclusion supported not only by the experimental results but also by the 2^3^ factorial design, which confirms that this mixture incorporates the optimal material proportions.

It is important to note that despite the exceptional properties of the best designs in this study, the results were found to be inferior when compared to similar previous investigations. This discrepancy can be attributed to the RA used in this study, which included significant microcracks caused by the mechanical processing of construction and demolition waste (CDW) and the presence of layers of “old” mortar. These factors affect the interfacial transition zone (ITZ) and the new cement paste. Specifically, the interaction zone between cement, sand (in some cases), and the aggregate results in a contact ITZ that, despite being larger, exhibits deficiencies and weaknesses.

In summary, the porosity of the M5 3/4” mixture proves to be optimal for the proper infiltration of rainwater. If this mixture were to be implemented in areas with light traffic—such as parks or recreational centers—it could generate significant favorable changes for the local flora and fauna. Furthermore, the porosity of these concrete pavements not only facilitates water infiltration and natural recharge of aquifers in densely populated urban areas but also effectively reduces noise levels. Therefore, their installation in parking areas or civic plazas could help mitigate acoustic pollution. Furthermore, its environmental impact is reduced by reusing construction and demolition waste, in line with global sustainability goals. Future research should build on these findings by investigating the long-term durability and maintenance requirements of RPC under various environmental conditions. There are different areas of opportunity, such as the integration of other complementary cementitious materials that could improve mechanical properties, as well as the analysis of the life cycle environmental and economic impacts of expanding the use of RPC in urban infrastructure projects.

## Figures and Tables

**Table 1:** Physic-mechanical properties of RA used for the different RPC blends [36,37,38,39,40].

Feature	Norm/Method	Acceptance Limit
Density	ASTM C 127-15	-
Compacted dry volumetric weight	ASTM C 29	-
Moisture absorption percentage	ASTM C 127-15	<5%
Percentage of wear and tear	ASTM C 131-06	<45%
	Micro-Deval	<30%
Granulometry	NMX-C-111-ONNCCE-2018	-

**Table 2:** Physical-mechanical properties of RA used.

Recycled Aggregate	Specific Gravity (g/cm^3^)	Absorption (%)	Compacted Dry Volumetric Weight (kg/m^3^)	Wear and Tear (%)	
				Los Angeles Test	Micro-Deval
3/8” RCA	2.23 ± 0.056	20.73 ± 12.670	1264.26 ± 16.79	35.62 ± 1.560	37.27 ± 2.350
3/8” RAP	2.32 ± 0.070	1.97 ± 0.239	1397.61 ± 8.470	33.51 ± 0.710	32.25 ± 1.220
3/4” RCA	2.32 ± 0.070	8.70 ± 1.706	1584.83 ± 16.770	25.20 ± 1.570	27.21 ± 1.560
3/4” RAP	2.35 ± 0.096	1.56 ± 0.375	1890.23 ± 2.910	29.34 ± 1.260	24.44 ± 1.970

Note: Average values ± Standard deviation. CDVW: Compacted Dry Volumetric Weight.

**Table 3:** Factors applied for the full factorial design 2^3^.

	Level	
Factor	Low (−1)	High (1)
Water/cement ratio (w/c)	0.26	0.35
Percentage of pores (%)	15	15
Percentage of fine aggregate (%)	0	10

**Table 4:** Full factorial design for the preparation of RPC mixtures.

Variables	W/C Ratio	Porosity (%)	Fine Aggregate (%)
1	0.26	15	0
2	0.35	15	0
3	0.26	20	0
4	0.35	20	0
5	0.26	15	10
6	0.35	15	10
7	0.26	20	10
8	0.35	20	10

**Table 5:** Composition of the different materials for the RPC mixtures.

3/8” RA Mixes					
Mix	RCA (kg)	RAP (kg)	Silica Sand (kg)	Cement (kg)	Water (*l*)
M1 3/8”	754.00	624.00	N/A	346.34	90.05
M2 3/8”	754.00	624.00	N/A	260.60	117.27
M3 3/8”	754.00	624.00	N/A	173.17	45.02
M4 3/8”	754.00	624.00	N/A	130.30	58.63
M5 3/8”	678.60	561.60	148.50	346.34	90.05
M6 3/8”	678.60	561.60	148.50	260.60	117.27
M7 3/8”	678.60	561.60	148.50	173.17	45.02
M8 3/8”	678.60	561.60	148.50	130.30	58.63
**3/4” RA Mixtures**					
M1 3/4”	724.75	754	N/A	346.34	90.05
M2 3/4”	724.75	754	N//A	260.60	117.27
M3 3/4”	724.75	754	N/A	173.17	45.02
M4 3/4”	724.75	754	N/A	130.30	58.63
M5 3/4”	652.27	678.60	148.50	346.34	90.05
M6 3/4”	652.27	678.60	148.50	260.60	117.27
M7 3/4”	652.27	678.60	148.50	173.17	45.02
M8 3/4”	652.27	678.60	148.50	130.30	58.63

N/A: Not applicable.

**Table 6:** Analysis of the factorial design of RPC mixtures with RA.

	Compressive Strength	Flexural Strength	Permeability	Volumetric Weight
Term (T)	Values (*p*)			
**3/8” RA mixes**				
Model	0.000	0.000	0.001	0.000
Lineal	0.000	0.000	0.000	0.000
W/C ratio	0.000	0.000	0.012	0.003
% Voids	0.000	0.000	0.000	0.000
% Fines	0.000	0.000	0.001	0.013
Interactions of 2 T	**0.319**	**0.241**	**0.144**	**0.197**
W/C ratio *% Voids	**0.163**	**0.055**	**0.927**	**0.080**
W/C ratio *% Fines	**0.329**	**0.792**	**0.398**	**0.254**
% Voids *% Fines	**0.432**	**0.855**	0.035	**0.554**
Interactions of 3 T	**0.527**	0.012	**0.648**	**0.705**
W/C ratio *% Voids *% Fines	**0.527**	0.012	**0.648**	**0.705**
	**R^2^** = 98.68%	**R^2^** = 96.63%	**R^2^** = 91.22%	**R^2^** = 94.38%
**3/4” RA mixtures**				
Model	0.000	0.000	0.002	0.000
Lineal	0.000	0.000	0.000	0.000
W/C ratio	0.000	0.000	0.005	0.003
% Voids	0.000	0.000	0.001	0.000
% Fines	0.000	0.000	0.002	**0.058**
Interactions of 2 T	0.000	0.028	**0.834**	**0.178**
W/C ratio *% Voids	0.000	0.031	**0.404**	**0.059**
W/C ratio *% Fines	0.000	0.038	**0.984**	**0.887**
% Voids *% Fines	0.000	**0.155**	**0.777**	**0.262**
Interactions of 3 T	0.000	0.001	**0.628**	0.034
W/C ratio *% Voids *% Fines	0.000	0.001	**0.628**	0.034
	**R^2^** = 99.53%	**R^2^** = 96.89%	**R^2^** = 89.41%	**R^2^** = 96.93%

## Data Availability

The original contributions presented in this study are included in the article. Further inquiries can be directed to the corresponding authors.
